# Artificial Intelligence‐Enhanced Quantitative 3D Analysis of Distal Radioulnar Ligament Insertion Footprints of the Triangular Fibrocartilage Complex With Interactive Validation

**DOI:** 10.1111/os.70231

**Published:** 2025-12-30

**Authors:** Zhe Yi, Wei Chen, Jiaxing Huang, Lei Zhu, Yantao Pei, Rebecca Qian Ru Lim, Lincoln Jian Rong Lim, Jia He, Yile Feng, Shuai Wang, Aijie Zhang, Weichen Wang, Ge Yang, Bo Liu

**Affiliations:** ^1^ Department of Hand Surgery, Beijing Jishuitan Hospital Capital Medical University Beijing China; ^2^ School of Artificial Intelligence University of Chinese Academy of Sciences Beijing China; ^3^ State Key Laboratory of Multimodal Artificial Intelligence Systems, CASIA Beijing China; ^4^ Dapartment of Hand Surgery Qilu Hospital of Shandong University Ji'nan China; ^5^ Department of Hand & Reconstructive Microsurgery Singapore General Hospital Singapore Singapore; ^6^ Department of Medical Imaging Western Health, Footscray Hospital Melbourne Australia; ^7^ Beijing Research Institute of Traumatology and Orthopaedics Beijing China

**Keywords:** artificial intelligence, image enhancement, magnetic resonance imaging, triangular fibrocartilage

## Abstract

**Objectives:**

The distal radioulnar ligaments (DRULs) serve as primary stabilizers to the distal radioulnar joint (DRUJ). Existing cadaveric studies report heterogeneous morphometric data of the three‐dimensional (3D) anatomy of the triangular fibrocartilage complex (TFCC) and the ulnar footprints of the DRULs due to methodological variations and small sample sizes, limiting the translation of precise anatomical knowledge to clinical practice. This study quantitatively evaluated the 3D anatomy of the TFCC and the insertions of both superficial and deep DRULs components using three different methods with subsequent interactive validation: (1) direct measurement, (2) 3D scan, and (3) artificial intelligence (AI) enhanced magnetic resonance imaging.

**Methods:**

Eleven adult cadaveric upper limbs were included. All specimens underwent 3.0‐Tesla MRI scans, which were then processed by AI algorithms for super‐resolution enhancement and semi‐automatic segmentation. The areas of deep and superficial limbs of DRUL ulnar footprint were measured in the super‐resolution MRI images using the Slicer software. The specimens were then dissected and anatomical measurements of dorsal‐volar maximal length and radial‐ulnar maximum length of deep ulnar DRUL footprint were performed on the specimens' photographs. Anatomical measurements of ulna, radius, triangular fibrocartilage, and ulnar insertions footprint of both superficial and deep DRULs were conducted subsequently using a 3D scanner. Primary outcome measures included the area and morphological classification (irregular quadrilateral, ribbon, semilunar) of the deep and superficial ulnar DRUL footprints. Statistical analysis encompassed intraclass correlation coefficients (ICC) for agreement assessment and multiple linear regression to explore associations.

**Results:**

The mean area of the deep foveal fibers of DRUL was 43.39 ± 13.49 mm^2^ and the superficial footprint was 20.11 ± 10.49 mm^2^ as measured with the 3D scanner. The morphologic features of the deep footprint shapes varied, with the most common shape being a ribbon (7/11, 64%). The intraclass correlation coefficients (ICCs) for the measurement of dorsal‐volar maximal length and radial‐ulnar maximum length of the DRUL between direct measurement and the 3D scan were excellent (ICC = 0.97 and 0.98, respectively). The ICCs between the AI‐enhanced analysis and the 3D scan for measuring the ulnar deep and superficial DRUL insertion areas were excellent (ICC = 0.95 and 0.96, respectively). Multiple linear regression explained 72.4% of the variance in deep DRUL footprint area (*R* ^2^ = 0.724, *p* = 0.147), with the superficial footprint area showing the strongest association (*β* = 0.639, *p* = 0.196).

**Conclusions:**

Compared to direct measurement and 3D scan, the AI algorithms developed and validated for wrist MRI image enhancement demonstrated high accuracy and reliability in anatomical measurements of DRULs.

## Graphical abstract

Interactive validation of a novel AI MRI super‐resolution and segmentation pipeline against direct anatomical measurement and 3D scanning for the precise quantification of distal radioulnar ligament (DRUL) insertion footprints.

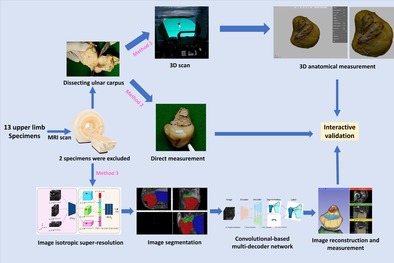

## 1 Introduction

The triangular fibrocartilage complex (TFCC) plays a critical role in stabilizing the distal radioulnar joint (DRUJ) [1, 2]. However, detailed anatomic knowledge of the TFCC, particularly the ulnar insertions of the distal radioulnar ligaments (DRULs), remains controversial. Accurate understanding of the TFCC insertion footprints is of great clinical significance, as it directly impacts the success of surgical repairs and the stability of the DRUJ [3]. Previous studies have shown that precise anatomical repair of the TFCC can improve clinical outcomes and reduce the risk of postoperative complications [4, 5, 6]. However, the discrepancies in current research findings can be attributed to the limited availability of cadaveric specimens, variations between specimens, the use of different measurement methods [7, 8, 9, 10], and the inherent uncertainties associated with manual measurements [10].

Utilizing cadaveric specimens, it is possible to obtain complete three‐dimensional (3D) structural data of critical wrist ligaments, and to create a 3D mapping of the important ligaments in the human wrist joint from the studied population. More importantly, digital anatomical data of real patients allows us to generate a digital twin of each patient's wrist that ultimately can be utilized for a variety of revolutionary digital‐assisted applications, including artificial intelligence (AI)‐aided diagnosis and robot‐assisted surgery [11, 12].

This study aims to quantitatively evaluate the 3D anatomy of the TFCC and specifically the insertions of DRULs using three methods (direct measurement, 3D scans, and AI‐enhanced wrist magnetic resonance imaging (MRI)) followed by interactive validation. The main goals of our study were to: (i) develop and validate a novel AI‐based algorithm for super‐resolution enhancement and automated segmentation of wrist MRI to enable precise 3D quantification of the TFCC, particularly the DRUL insertion footprints; (ii) provide detailed, quantitative anatomic data of the ulnar insertion footprints of the superficial and deep DRULs, including their area, morphology, and topographic relationship to the distal ulna and radius; (iii) establish the foundation for a patient‐specific “digital twin” model of wrist anatomy derived from clinical MRI, with potential applications in AI‐aided diagnosis, preoperative planning, and robot‐assisted surgery.

## 2 Methods

This was a cadaveric study and was approved by the Ethics Committee of Beijing Jishuitan Hospital (No. K2024‐024‐01).

### 2.1 Specimen Preparation

A total of 13 adult fresh frozen upper limb specimens (seven left and six right limbs) from eight cadavers with no bony deformity or severe arthritis were initially included in this study. The donors consisted of five males and three females and the mean age at the time of death was 69 years (range, 52–85 years). All wrists were radiographically imaged and demonstrated normal osseous anatomy. Specimens demonstrating age‐related alterations in the TFCC were included on the condition that both the superficial and deep components of the DRULs remained intact. Exclusion criteria included degeneration changes or tears of the DRULs. Two specimens were excluded when obvious degeneration in the proximal ligament of the deep DRUL on the ulnar side was detected on MRI.

### 2.2 MRI Scan, Super‐Resolution, Segmentation, Reconstruction, and Visualization

MRI scans were performed in all 13 upper limb specimens with an Ingenia 3.0‐T scanner (Philips Healthcare, Amsterdam, The Netherlands) combined with an eight‐channel wrist coil in our institution. All subjects underwent MRI examination with the same sequences: proton density fat‐saturated imaging in coronal, sagittal and axial planes; and T1‐weighted fast spin‐echo imaging in the coronal plane. All subjects underwent thin‐slice MRI scanning. The slice thickness was 1 mm, with no interslice gap. The field of view was 90 × 90 mm. The total scan time was about 15 min for one wrist.

**FIGURE 1:**
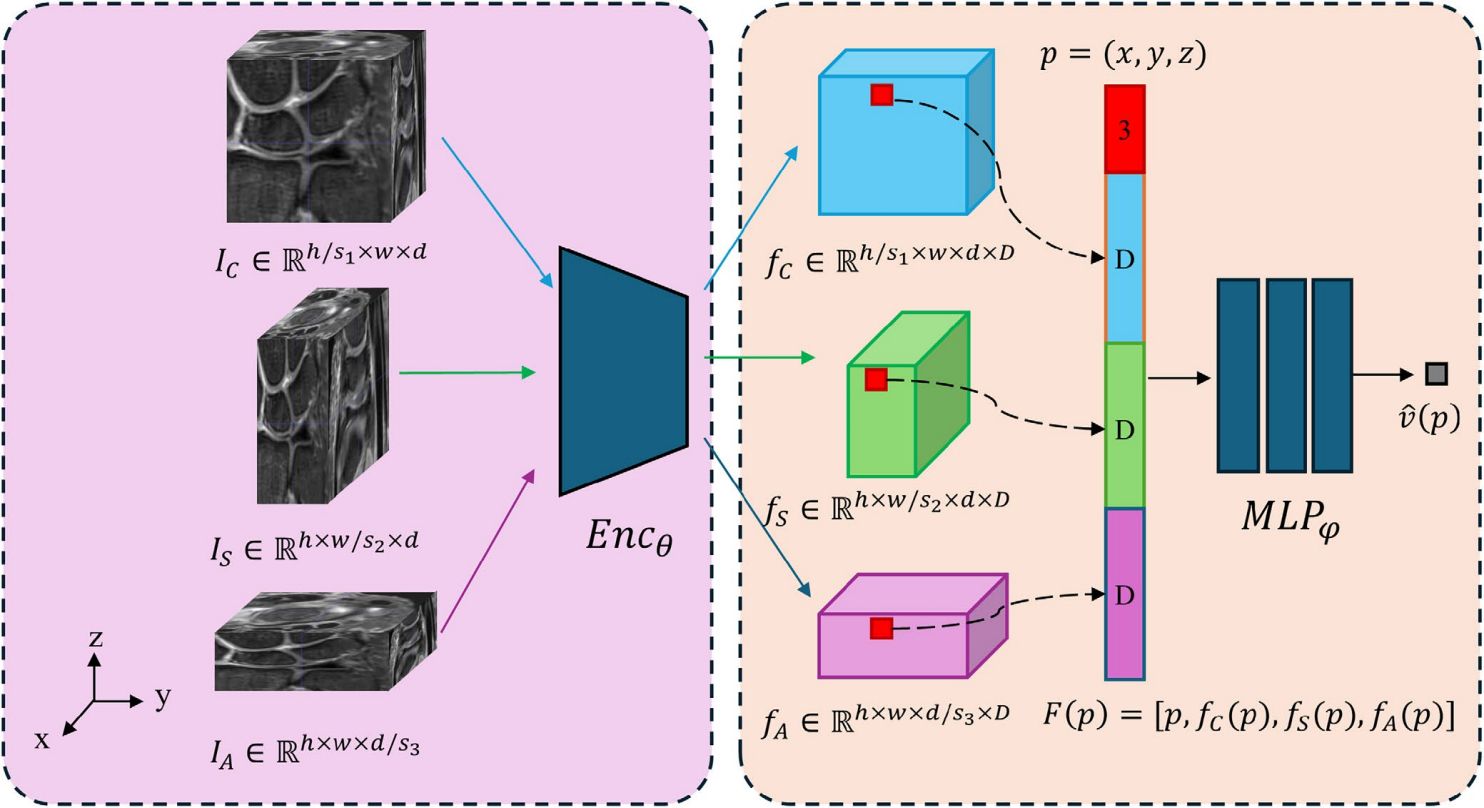
Enhancement of MRI images by implicit neural representation (INR)–based multiple‐volume isotropic super‐resolution.

We propose a multi‐volume MRI super‐resolution method for reconstructing a single isotropic high‐resolution image by integrating three orthogonally acquired anisotropic‐resolution 3D MRI volumes (coronal, sagittal, and axial views) [13]. The algorithm has been granted an invention patent (Chinese Patent No. ZL 2024 1 0393914.2) but is currently not open source at this stage (Figure 1). Our approach employs an implicit neural field technique to represent 3D medical images as continuous neural representations, enabling arbitrary resolution up sampling. By leveraging a two‐dimensional Fourier spectral loss function, the proposed method effectively integrates high‐frequency signals from the in‐plane slices of low‐resolution volumes while mitigating blurring artifacts along the through‐plane direction. This strategy circumvents the spatial averaging effects often observed in other multi‐view fusion approaches. Notably, our method operates in a fully self‐supervised training manner without requiring isotropic ground truth data for model training. In terms of implementation, the model was pretrained on 121 sets of wrist MRI volumes from clinical patients and evaluated on 24 independent cadaveric wrist MRI volumes from a prior, unrelated study, achieving approximately 11‐fold isotropic super‐resolution (from 0.1 × 0.1 × 1.1 mm^3^ to 0.1 × 0.1 × 0.1 mm^3^). All clinical MRI data were obtained retrospectively with institutional review board approval and waiver of informed consent. The cadaveric MRI data used for evaluation were derived from a separate specimen cohort, distinct from the 11 specimens analyzed in the main morphometric study of this work. The reconstructed volumes exhibit superior anatomical fidelity with enhanced boundary clarity and reduced artifacts, achieving isotropic reconstruction for real wrist MRI volumes and facilitating downstream assessment of TFCC.

After performing super‐resolution reconstruction on MRI images, we employed ITK‐SNAP (version 3.0) to construct a 3D dataset through a two‐step process of rough segmentation followed by refinement, resulting in a dataset comprising 70 training, 10 validation, and 20 test cases. Subsequently, based on this dataset, we proposed a convolution‐based multi‐decoder network integrated with Slicer software (version 5.6.2) to facilitate the segmentation, reconstruction, and visualization of the following anatomical structures: the radius, ulna, TFCC, and the ulnar insertion region of the DRULs.

**FIGURE 2:**
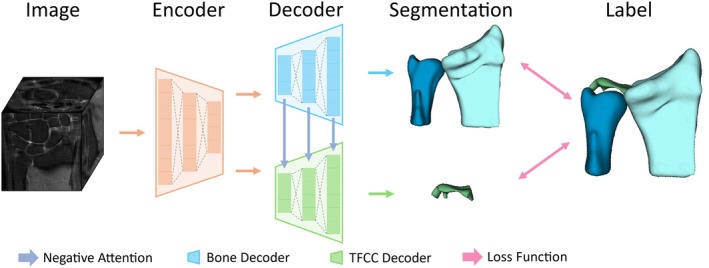
Architecture of proposed convolution‐based multi‐decoder segmentation network.

As illustrated in Figure 2, two clinical prior knowledge components were systematically incorporated into the model architecture to enhance anatomical segmentation accuracy: (1) the distinct intensity distributions between bone structures (radius/ulna) and the TFCC, and (2) their differential volumetric characteristics. The architectural design addresses these anatomical differences through two specialized pathways: a Bone Decoder optimized for large‐volume bone segmentation and a TFCC Decoder tailored for thin cartilage structures. To leverage the segmentation advantage of larger osseous structures, we implemented a negative attention mechanism in which bone decoder features guide TFCC feature refinement through adaptive gating, thereby improving precision in challenging TFCC segmentation.

With these enhancements, our model achieves DICE scores (see Equation (1) below) of 0.92 for radius and ulna segmentation and 0.81 for TFCC segmentation. Given an image I with N pixels, the segmentation ground truth is denoted as y, where foreground and background pixels are labeled as 1 and 0, respectively. The prediction of the model is represented as $\hat{y\;}\epsilon \;\left[0,1\right]$, denoting the probabilities of individual pixels being classified as foreground. The Dice score is defined as:

(1) $$\text{Dice}=\frac{2\sum_{i=1}^{c}{\hat{y\;}}_{\mathrm{ii}}}{\sum_{i=1}^{c}\sum_{j=1}^{c}{\hat{y\;}}_{\mathrm{ij}}+\sum_{i=1}^{c}\sum_{j=1}^{c}{\hat{y\;}}_{\mathrm{ji}}}$$

where c is the number of segmentation classes. The upper limit of Dice scores is 1. The following differentiable Soft IoU loss was used in training:

(2) $$L_{\mathrm{iou}}=1-\frac{\sum_{i=1}^{N}y_{i}\hat{y_{i}}\;}{\sum_{i=1}^{N}y_{i}+\hat{y_{i}}-y_{i}\hat{y_{i}}+\eta }$$

where η is a smoothing coefficient.

**FIGURE 3:**
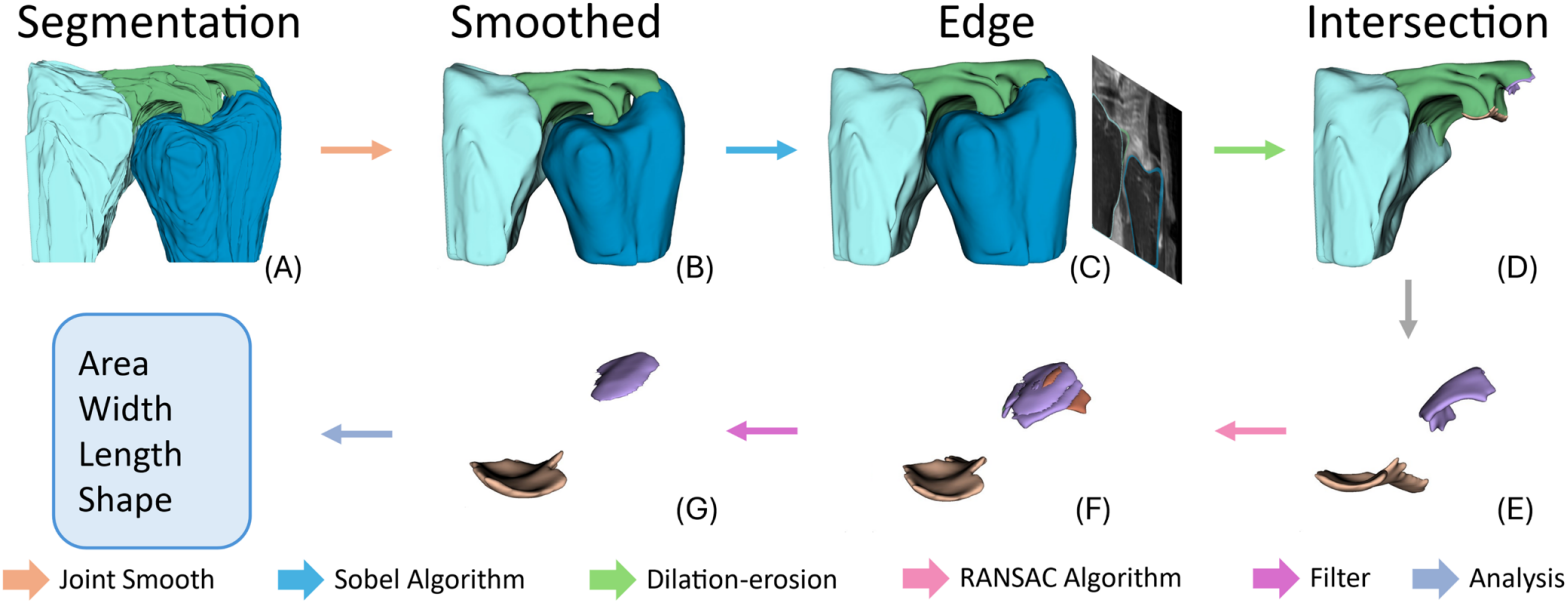
Workflow of MRI image reconstruction, visualization, and quantitative analysis by our AI algorithms and the Slicer software.

To extract the TFCC insertion footprints, we developed an integrated processing pipeline as shown in Figure 3. The workflow starts with deep learning‐based image segmentation, followed by joint smoothing optimization. Sobel edge detection is used then to delineate boundaries of TFCC, radius, and ulna respectively. Subsequent morphological operations (dilation‐erosion) identify the TFCC‐ulnar overlapping region as the preliminary footprint. Then, prior to quantitative analysis, the footprint regions are refined using the RANSAC plane fitting algorithm [14], which iteratively optimizes inlier points to establish articular planes and selects the maximum continuous area. Finally, based on the refined results, data analysis is performed to obtain metrics such as area, length, width, and shape characteristics. In the Slicer software, visualization of the other components of TFCC is temporarily switched off so that only the ulnar footprints of DRUL area connected to the ulnar head and ulnar styloid are visualized. Areas of deep and superficial limbs of DRUL ulnar footprint are measured in the Slicer software.

### 2.3 Specimen Anatomy Dissections

All soft tissues were removed except for the TFCC and DRUJ capsules after MRI scanning. To observe the gross distribution of the ulnar RUL insertions, we opened the DRUJ in all the specimens, detached the radial origins of the TFCC on the sigmoid notch, resected the distal radius, and disarticulated the radiocarpal joint. The non‐ligamentous tissues were dissected carefully and removed from the ulnar fovea and styloid process to allow the deep and superficial limbs of the RUL to be distinguished clearly. The boundary of the ulnar footprint of DRUL was outlined by a black marker pen. Based on the outline of the footprint of the deep DRUL, the shape of the deep DRUL was classified into three types: irregular quadrilateral, ribbon, and semilunar [7]. We used a camera to photograph the specimen, and a 0.5 cm ruler was placed on the head of the ulna. The dorsal‐volar and radial‐ulnar maximum length of the TFCC ulnar deep footprint was measured with calibration by the ruler.

### 2.4 3D Scan and Anatomical Measurement

**FIGURE 4:**
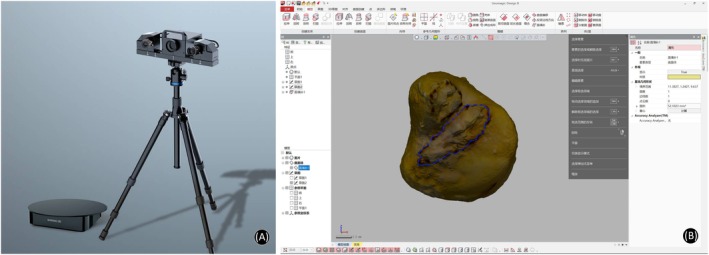
Equipment for 3D scan and software for anatomical measurement. (A) A snapshot of the Transcan C 3D scan. (B) A snapshot of the user interface of the Geomagic Design X software.

**FIGURE 5:**
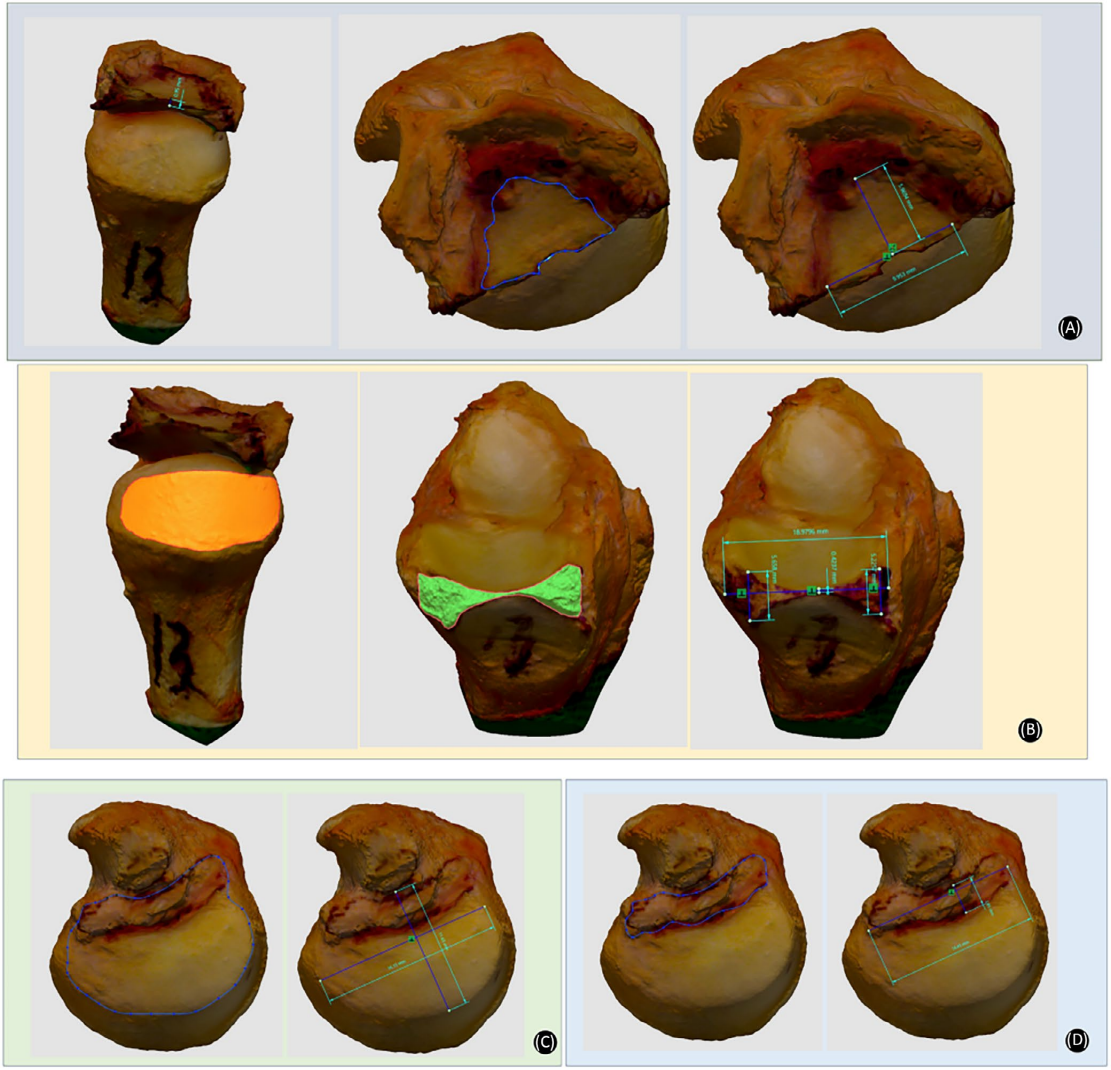
Anatomical 3D data measured in Geomagic Design X software. (A) Center thickness, area, DV, and RU maximum length of TFC. (B) Sigmoid notch area of the ulna; center thickness, DV maximum length, and area of sigmoid notch of the radius. (C) DV and RU maximum length, and area of ulna head. (D) DV and RU maximum length, and area of ulnar footprint of deep DRUL. Area of ulnar footprint of superficial DRUL.

To accurately depict the 3D data of TFCC, ulna, and radius, we used a commercial 3D scanner (Transcan C, Shining 3d Tech Co. Ltd., Hangzhou, Zhejiang Province, China; Figure 4A) with “variable resolution” based on high‐precision 3D digitization technology to scan the specimens. The scanner software recorded the 3D coordinates of any selected areas relative to a coordinate system created by reference points. Distances between points and areas of irregular shapes can then be calculated. The captured 3D scan data then were imported to the Geomagic Design X software (3D systems, Rock Hill, SC, USA; Figure 4B) to precisely measure the anatomical data, which include: center thickness, area, dorsal‐volar, and radial‐ulnar maximum length of Triangular fibrocartilage (TFC); sigmoid notch area of the ulna; dorsal‐volar and proximal‐distal maximum length, center thickness, and area of sigmoid notch of the radius; dorsal‐volar and radial‐ulnar maximum length and area of ulna head; dorsal‐volar and radial‐ulnar maximum length and area of ulnar footprint of deep DRUL limb (Figure 5).

### 2.5 Statistical Analysis

For quantification, the mean and standard deviation (SD) of measurements were calculated. Unless specified otherwise, measurement results shown in the form of X ± Y indicate mean X ± SD Y.

Statistical analysis was performed using SPSS software (version 25 for Windows, Chicago, IL, USA). Multiple linear regression was employed to examine relationships between deep ulnar DRUL footprint area and six selected anatomical parameters, including “superficial ulnar DRUL footprint area,” “ulna head area,” “area of the sigmoid notch of the radius and ulna,” “TFC center thickness,” and “TFC area.” Assumptions were verified through residual analysis and variance inflation factors. Normality was assessed via Q–Q plots. Model fit was evaluated using *R* ^2^, adjusted *R* ^2^, and F‐statistics. All tests were two‐sided with significance at *p* < 0.05.

Intraclass correlation coefficient (ICC) was used to evaluate reliability and consistency between different measurements. The ICC was used to determine the agreement levels, which were classified as poor (ICC < 0.5), moderate (ICC 0.5–0.75), good (ICC 0.75–0.9), and excellent (ICC > 0.9).

A post hoc statistical power analysis was conducted using G*Power software (version 3.1, Heinrich‐Heine‐Universität Düsseldorf, Düsseldorf, Germany) to quantify the probability of detecting the observed effect size. Given the sample size (*n* = 11), number of predictors (*k* = 6), and an observed effect size of *f* ^2^ = 2.62 (derived from *R* ^2^ = 0.724), the analysis indicated a statistical power of 59% at an α level of 0.05.

## 3 Results

### 3.1 Quantitative Measurements of the 11 Specimens

#### 3.1.1 Direct Measurement

Morphologic features of the deep footprint shapes were ribbon in 7, irregular quadrilateral in 3, and semilunar in 1 out of the 11 upper limb specimens.

The dorsal‐volar (DV) maximal length and radial‐ulnar (RU) maximum length, measured through direct measurement on the specimens' photographs of the deep DRUL, were 13.34 (SD 2.24) and 3.93 (SD 1.07) mm, respectively.

#### 3.1.2 3D Scan Data

The anatomical data of TFC, ulna head, sigmoid notch of the ulna and radius, ulnar footprint of deep DRUL measured through 3D scan were shown in Table 1. The center thickness, DV maximal length, RU maximum length, and area of the TFC were 0.60 (SD 0.16) mm, 11.37 (SD 1.47) mm, 7.68 (SD 1.25) mm, 69.58 (SD 17.55) mm^2^, respectively. The area of the sigmoid notch of the ulna was 134.42 (SD 32.96) mm^2^. The center thickness, DV maximal length, and area of the sigmoid notch of the radius were 0.86 (SD 0.23), 17.47 (SD 1.67) mm, and 63.12 (SD 8.60) mm^2^, respectively. The DV maximal length, RU maximum length, and area of the ulna head were 16.12 (SD 2.65) mm, 10.49 (SD 1.67) mm, and 154.82 (SD 40.75) mm^2^, respectively. The DV maximal length, RU maximum length, and area of the footprint of the deep DRUL were 13.4 (SD 2.31), 3.98 (SD 1.06) mm, and 43.39 (SD 13.49) mm^2^, respectively. The area of the footprint of the superficial DRUL was 20.11 (SD 10.49) mm^2^.

**TABLE 1:** TFC, ulna head, sigmoid notch of the ulna and radius, ulnar footprint of deep DRUL anatomical data were measured in Transcan C.

Structures	Parameters	Mean ± SD (mm/mm^2^)
TFC	Center thickness	0.60 ± 0.16
	DV maximum length	11.37 ± 1.47
	RU maximum length	7.68 ± 1.25
	Area	69.58 ± 17.55
Sigmoid notch of the ulna	Area	134.42 ± 32.96
Sigmoid notch of the radius	Center thickness	0.86 ± 0.23
	DV maximum length	17.47 ± 1.67
	Area	63.12 ± 8.60
Ulna head	DV maximum length	16.12 ± 2.65
	RU maximum width	10.49 ± 1.67
	Area	154.82 ± 40.75
Deep ulnar footprint of DRUL	DV maximum length	13.40 ± 2.31
	RU maximum width	3.98 ± 1.06
	Area	43.39 ± 13.49
Superficial ulnar footprint of DRUL	Area	20.11 ± 10.49

Abbreviations: DRUL, distal radioulnar ligament; DV, dorsal‐volar; RU, radial‐ulnar; TFC, triangular fibrocartilage.

### 3.2 AI Reconstruction Data

Ulnar footprints of both deep and superficial DRUL anatomical data were measured in the Slicer software and shown in Table 2. The areas of the deep and superficial footprint of DRUL were 43.66 (SD 12.72) and 20.98 (SD 10.14) mm^2^, respectively.

**TABLE 2:** Ulnar footprint of both deep and superficial DRUL anatomical data measured in Slicer software using the AI algorithms developed for this study.

Structures	Parameters	Mean ± SD (mm^2^)
Ulnar footprint of deep DRUL	Area	43.66 ± 12.72
Ulnar footprint of superficial DRUL	Area	20.98 ± 10.14

### 3.3 Correlations Between the Deep Ulnar DRUL Footprint Area and Distal Ulna and Radius Topography

Multiple linear regression was employed to investigate the relationship between the deep ulnar DRUL footprint area and the morphology of the distal ulna and radius, with six parameters as independent variables: the superficial ulnar DRUL footprint area, ulnar head area, radial and ulnar sigmoid notch areas, TFCC central thickness, and TFCC area. The regression model explained 72.4% of deep DRUL area variance (*R* ^2^ = 0.724), though adjusted *R* ^2^ was 0.448, suggesting overfitting. The overall model was non‐significant (*F* = 2.624, *p* = 0.147). Superficial ulnar DRUL footprint area showed the strongest association trend (*β* = 0.639, *p* = 0.196), while other parameters demonstrated non‐significant effects. Variance inflation factors (2.135–3.366) indicated acceptable multicollinearity.

### 3.4 Results of Consistency Test

The ICC for the measurement of DV maximal length and RU maximum length of the DRUL between direct measurement on the specimens' photographs and 3D scan was excellent, at 0.97 and 0.98, respectively. The ICC for the measurement of deep and superficial ulnar footprint area of the DRUL between the 3D scan method and AI algorithms was also excellent, at 0.95 and 0.96, respectively.

## 4 Discussion

Accurate understanding of the footprint of the DRULs is of great importance [7, 11]. However, the published research findings to date are varied and exhibit discrepancies. The reasons for these discrepancies include the limited number of available specimens, variations between specimens, and different methods used for measurement.

### 4.1 Summary of Key Findings

This study conducted an in‐depth quantitative analysis of the anatomy of the TFCC and its related structures by combining 3D scan technology and an AI‐based MRI super‐resolution enhancement algorithm. The results revealed the detailed 3D morphology of the DRUL and its ligament attachment components, which are of great significance for understanding the stability mechanisms and biomechanical properties of the DRUJ.

#### 4.1.1 Validation of the AI Algorithm and Key Anatomic Insights

The center of the ulna fovea is considered as an isometric point of the RUL during DRUJ rotation according to previous studies [15, 16]. However, our results showed that ribbon‐shaped footprints account for the largest proportion (7/11, 64%) of the ulnar footprint of the deep DRUL, which was different from the 32% ribbon shape proportion as reported by Zhao et al. [7]. Diverse morphologies of the deep DRUL footprint were observed, including irregular quadrilateral, ribbon, and semilunar shapes in our study, supporting Zhao's viewpoint on the complexity and diversity of the ulnar DRUL footprint anatomical structure [7]. The observed variations could be attributable to the heterogeneous sources of the specimens and the limited sample size, both of which were potential sources of bias. Our morphometric analysis delineates the deep ulnar DRUL as a broad, ribbon or semilunar‐shaped footprint, nearly spanning from the palmar to the dorsal aspect of the ulnar head. The center of the deep DRUL footprint was located on the radial wall rather than the bottom of the fovea. We speculate that isometric repair of the deep RUL at the foveal center may be inappropriate and may result in abnormal DRUJ kinematics. Our findings support targeted anatomical repair techniques which reattach the volar and dorsal bands of DRULs, thereby reconstructing its broad foveal insertion. Further biomechanical and clinical studies are needed to test and verify these concepts.

Our research results also showed that the footprint area of the foveal fibers of the DRUL is relatively large, with an average area of 43 mm^2^, as shown in Table 3, which is more than expected upon comparison with previous studies (Shin et al.'s study [9] reported 29.7 mm^2^ and Maniglio et al.'s study [8] reported 31.5 mm^2^). Apart from ethnicity and other intrinsic factors of various specimens, the difference may be due to the use of more precise 3D scanning and AI algorithms in this study, which improved the accuracy and reliability of the data.

**TABLE 3:** Summary of reported DRUL ulnar footprint morphometry, 2020–2024.

Author (year)	Number of specimen	Footprint of DRUL on ulna				
		Measuring technique and method	Deep footprint shape	Deep footprint length * width (mm)	Deep footprint area (mm^2^)	Superficial footprint area (mm^2^)
Shin et al. [9]	13	Micro‐CT imaging; Painted with Telebrix		12.68 * 3.10	29.67	18.36
Maniglio et al. [8]	21	MicroScribe 3DLX; Labeling ligaments with ink	Semilunar	9.3 * 1.8	31.5	10.6
Okuda et al. [10]	26	Computed tomography & Mimics; Ligament boundary marked by 1.0‐mm drill		9 * 6	34	
Zhao et al. [7]	25	Microscope; Outlined by a black marking pen	Irregular quadrilateral (28%), ribbon (32%), semilunar (40%)	8.4 * 3.7	26.3	
Our study	11	Transcan C & MRI AI enhancement algorithm; Outlined by a black marking pen	Irregular quadrilateral (27%), ribbon (64%), semilunar (9%)	13.40 * 3.98	43.39	20.98

#### 4.1.2 Quantitative Relationships From Multivariate Analysis

While our regression model explained a substantial portion of the variance in the deep footprint area (*R* ^2^ = 0.724), the associations did not reach statistical significance, precluding definitive conclusions regarding its correlation with specific bony dimensions. Consequently, the postulated link between smaller bone size and increased susceptibility to TFCC injury, while biomechanically plausible, remains speculative and is not directly supported by our statistical model. These preliminary observations highlight a compelling area for future research with larger cohorts, powered to robustly determine the morphometric factors influencing TFCC anatomy and its potential relationship to injury risk.

#### 4.1.3 Clinical Implications and the Path to Patient‐Specific Modeling

Our study found that the footprint area of the superficial DRUL is relatively small, with an average of only 20 mm^2^. Recent studies have indicated that instability of the DRUJ did not fully correlate with ulnar styloid fractures [17, 18, 19]. Meta‐analyses have shown that further intervention is only necessary in cases of distal radius fractures accompanied by ulnar styloid base fractures combined with DRUJ instability [18]. Zhao et al. [7] did not find any significant ligamentous tissue attachment to the base of the ulnar styloid. Our study results demonstrate that the footprint area of the DRUL at the ulnar styloid accounts for less than 50% of the area of the deep DRUL fovea insertion area. This finding is consistent with previous research [8, 9]. The superficial component of the DRUL plays a less significant role in DRUJ stability compared to the deep component. This discovery emphasizes the important role of the deep DRUL in maintaining DRUJ stability and suggests that special attention should be paid to preserving the integrity of the deep DRUL during TFCC‐related surgeries to avoid adverse effects on DRUJ stability.

Notably, Rollo et al. demonstrated that bone grafting combined with the Sauvé‐Kapandji procedure effectively treated aseptic distal radius non‐union, achieving full fracture union and improving DRUJ‐related functions (e.g., wrist motion, grip strength) [20]. Since this procedure targets DRUJ balance restoration, our DRUL anatomical data can provide critical preoperative references to optimize ligament/osseous alignment, complementing its clinical efficacy.

### 4.2 Toward Patient‐Specific Digital Twins: Technical Validation and Clinical Potential of AI‐Enhanced Wrist MRI

**FIGURE 6:**
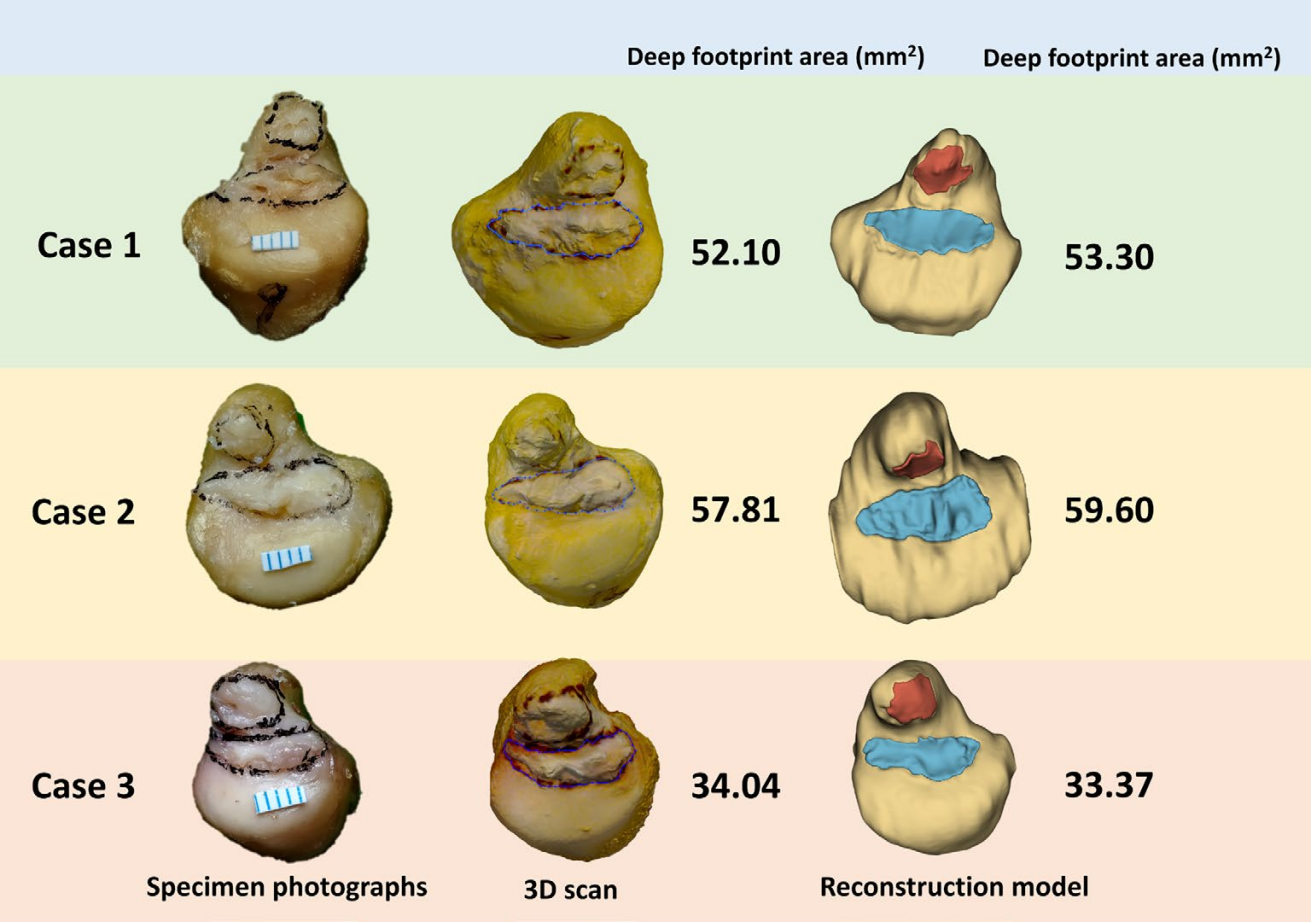
Three examples of 3D scan and AI enhancement algorithm in measuring the area of deep footprint of DRUL in ulna fovea.

While MRI can effectively display the ligamentous structures of large human joints like the knee and shoulder [21, 22, 23], and AI‐based algorithms can provide digital information of important anatomical structures of these large joints to address the above‐mentioned drawbacks of cadaveric studies [24, 25], there is currently a lack of research focusing on small and intricately structured joints like the wrist joint. This study implemented the first full process of super‐resolution, segmentation, reconstruction, and visualization of MRI images using AI algorithms, enabling clearer observation and analysis of the anatomical structure of the TFCC and its related components in virtual images. By comparing the measurement results of the AI algorithms with 3D scan and direct measurement on the specimens' photographs (Figure 6), we validated the accuracy and reliability of the AI algorithms in anatomical measurements. This study demonstrates that the novel AI‐enhanced wrist MRI algorithm exhibits comparable accuracy and consistency to 3D scan and measurement methods on specimens. This not only holds the potential to analyze wrist MRI scans from a large cohort of healthy volunteers in the future, thereby obtaining accurate anatomical data of wrist ligaments from a substantial population and paving the way for creating a 3D mapping of the important ligaments in the human wrist joint, but more importantly, the anatomical data of each individual wrist joint obtained through this new AI algorithm represents a digital twin of that individual. Different from the previous purely anatomical measurement method, this new method allows us to generate a digital twin of each patient's wrist anatomy based on their MRI in the future. These digital twins can then be directly utilized for a variety of revolutionary digital‐assisted clinical applications, including AI‐aided diagnosis and robot‐assisted precision treatment.

### 4.3 Strengths and Limitations

The primary strengths of this study lie in its integrative and validated methodological approach. First, we employed a unique triad of methods—direct anatomical measurement, high‐precision 3D scanning, and a novel AI‐enhanced MRI algorithm—for cross‐validation, significantly enhancing the robustness of the morphometric data. Second, to our knowledge, this is the first study to successfully apply and validate a dedicated AI super‐resolution and segmentation algorithm for the detailed 3D quantification of the ulnar insertion footprints of DRULs from clinical‐grade wrist MRI, bridging a critical gap between cadaveric anatomy and in vivo clinical imaging.

This study also has several limitations. First, the sample size of cadaveric specimens is somewhat limited. However, it is comparable to those in similar studies, and the results obtained from our two methods exhibited a high degree of consistency. Second, a post hoc power analysis revealed a statistical power of only 59%, well below the conventional 80% threshold. Consequently, our study had a limited capacity to detect true associations, increasing the risk of Type II errors. Therefore, the non‐significant *p*‐values should be interpreted with caution, and the substantial effect size (*R* ^2^ = 0.724) must be considered preliminary. Future studies with larger sample sizes are essential to corroborate these potential relationships with adequate statistical power. Additionally, we focused on numerical area comparisons rather than direct 3D morphological assessments of the foveal insertion area of the DRUL among the methods. This may have affected the precision of our results to some extent.

## 5 Conclusions

In summary, this study provided an in‐depth quantitative analysis of the anatomical characteristics of the TFCC, particularly the ulnar insertions of the DRULs. Ribbon‐shaped footprint accounts for the largest proportion of the ulnar footprint of the deep DRUL. The AI algorithms developed and validated in this study for super‐resolution enhancement of MRI images offer high accuracy and reliability in anatomical measurements of these critical wrist structures. This novel method not only enables the analysis of wrist MRI scans from large cohorts to create accurate anatomical data and 3D mapping of wrist ligaments but also, more importantly, holds the potential to generate digital twins of individual wrist anatomy, enabling revolutionary digital‐assisted applications, such as AI‐aided diagnosis and robot‐assisted surgery.

## Author Contributions

All authors had full access to the data in the study and take responsibility for the integrity of the data and the accuracy of the data analysis. Conceptualization: Bo Liu and Ge Yang. Methodology: Zhe Yi, Wei Chen, Jiaxing Huang, Jia He, Yile Feng, Rebecca Qian Ru Lim, and Lincoln Jian Rong Lim. Validation: Zhe Yi, Wei Chen, and Jiaxing Huang. Investigation: Zhe Yi, Wei Chen, Shuai Wang, Weichen Wang, Aijie Zhang, Yantao Pei, and Lei Zhu. Formal analysis: Zhe Yi, Wei Chen, and Jiaxing Huang. Resources: Bo Liu, Yantao Pei, and Lei Zhu. Original draft: Zhe Yi. Review and editing: Zhe Yi, Wei Chen, Jiaxing Huang, Bo Liu, Ge Yang, Rebecca Qian Ru Lim, and Lincoln Jian Rong Lim. Visualization: Zhe Yi and Jiaxing Huang. Supervision: Bo Liu and Ge Yang. Funding acquisition: Bo Liu. We confirm that all authors listed meet the authorship criteria according to the latest guidelines of the International Committee of Medical Journal Editors, and that all authors are in agreement with the manuscript.

## Funding

This study was supported by the National Natural Science Foundation of China (82272581), the Beijing Natural Science Foundation‐Haidian Original innovation Joint Fund Project (L252124), the Beijing Hospitals Authority's Ascent Plan (DFL20240402), and the Beijing Municipal Health Commission (BJRITO‐RDP).

## Disclosure

The authors have nothing to report.

## Ethics Statement

This study was approved by the Institutional Ethics Review Board of Beijing Jishuitan Hospital (IRB: K2024‐024‐01). All methods were performed in accordance with the 1964 Declaration of Helsinki and its later amendments, and the ethical standards of the institutional research committee.

## Consent

The authors have nothing to report.

## Conflicts of Interest

The authors declare no conflicts of interest.

## Data Availability

The data that support the findings of this study are available from the corresponding author upon reasonable request.
